# Knowledge, attitudes, and practices related to antibiotic use in Paschim Bardhaman District: A survey of healthcare providers in West Bengal, India

**DOI:** 10.1371/journal.pone.0217818

**Published:** 2019-05-31

**Authors:** Mohit Nair, Santanu Tripathi, Sumit Mazumdar, Raman Mahajan, Amit Harshana, Alan Pereira, Carolina Jimenez, Debasish Halder, Sakib Burza

**Affiliations:** 1 Medecins Sans Frontieres, New Delhi, India; 2 Calcutta School of Tropical Medicine, Kolkata, India; 3 Medecins Sans Frontieres, Barcelona, Spain; 4 Paschim Bardhaman Health District, West Bengal, India; University of Campania, ITALY

## Abstract

**Introduction:**

Antibiotic misuse is widespread and contributes to antibiotic resistance, especially in less regulated health systems such as India. Although informal providers are involved with substantial segments of primary healthcare, their level of knowledge, attitudes, and practices is not well documented in the literature.

**Objectives:**

This quantitative study systematically examines the knowledge, attitudes, and practices of informal and formal providers with respect to antibiotic use.

**Methods:**

We surveyed a convenience sample of 384 participants (96 allopathic doctors, 96 nurses, 96 informal providers, and 96 pharmacy shopkeepers) over a period of 8 weeks from December to February using a validated questionnaire developed in Italy. Our team created an equivalent, composite KAP score for each respondent in the survey, which was subsequently compared between providers. We then performed a multivariate logistic regression analysis to estimate the odds of having a low composite score (<80) based on occupation by comparing allopathic doctors (referent category) with all other study participants. The model was adjusted for age (included as a continuous variable) and gender.

**Results:**

Doctors scored highest in questions assessing knowledge (77.3%) and attitudes (87.3%), but performed poorly in practices (67.6%). Many doctors knew that antibiotics were not indicated for viral infections, but over 87% (n = 82) reported prescribing them in this situation. Nurses, pharmacy shopkeepers, and informal providers were more likely to perform poorly on the survey compared to allopathic doctors (OR: 10.4, 95% CI 5.4, 20.0, p<0.01). 30.8% (n = 118) of all providers relied on pharmaceutical company representatives as a major source of information about antibiotics.

**Conclusions:**

Our findings indicate poor knowledge and awareness of antibiotic use and functions among informal health providers, and dissonance between knowledge and practices among allopathic doctors. The nexus between allopathic doctors, pharmaceutical company representatives, and informal health providers present promising avenues for future research and intervention.

## Introduction

Antibiotics have been a crucial development in the evolution of medical treatment, effectively reducing the morbidity and mortality from bacterial diseases that were previously left untreated [1]. However, irrational use of antibiotics (including veterinary antimicrobial misuse/overuse, environmental contamination, nosocomial transmission, suboptimal point of care diagnostics, and suboptimal dosing) has contributed to the emergence and selection of resistant bacteria [2]. Consequently, World Health Organization (WHO) has warned that the world is entering a "post antibiotic" era where even minor infection and injury, previously manageable with antimicrobials, will cost lives [3].

It is well known that knowledge of antibiotics is quite poor among patients and the general public in multiple countries [4–10]. Among healthcare providers, antibiotics are routinely prescribed for acute upper respiratory tract infections [11–13], despite clinical evidence to the contrary. The misuse of antibiotics is particularly striking in India, which is ranked as one of the world’s largest consumers of antibiotics for human health [14]. Most private pharmacies in India are operated by unqualified persons rather than trained and licensed pharmacists, which exacerbates the practice of disbursing medicines normally not available over-the-counter without a prescription from an appropriate medical practitioner. The Drugs & Cosmetics Act and Rules provide regulatory enforcement to the sale of antimicrobials in India, as well as a mandate to identify unlicensed pharmacies, and unqualified medical practitioners and prescribers [15]. The rules state that only qualified medical practitioners can prescribe medicine, but this is often not the case as many informal health providers (IHPs), who do not hold formal medical degrees and are untrained in allopathic medicine, disburse antibiotics as part of their regular practice [16].

However, there is limited evidence regarding the perceptions and drivers of antibiotic misuse in the Indian setting [17–21], and there are no published studies focusing on the Knowledge, Attitudes and Practices (KAP) of antibiotic use from West Bengal. We were unable to find any recent studies that comprehensively assess the KAP of formal and informal providers alike in relation to antibiotic use in India. Understanding local knowledge, attitudes and practices regarding antibiotics will be the first step in constructing a template for effective antibiotic stewardship and effective infection control measures. Such a template is essential in planning future interventions for antibiotic resistance throughout India.

This study addresses these gaps in the literature by systematically examining the prescribing patterns of informal and formal providers, and assessing how informal providers differ from allopathic doctors in knowledge, attitudes and practices in Paschim Bardhaman district, located in the state of West Bengal, India.

## Materials and methods

A self-administered 38-question KAP survey tool was provided to allopathic doctors, informal healthcare providers, nurses, and pharmacy shopkeepers, in order to establish the knowledge, attitudes, and practices related to antibiotic use in Paschim Bardhaman district of West Bengal. Appropriate antibiotic use was defined as the right antibiotic at the right time, in the right dose, and for the right duration [22]. The questionnaire was adapted from a validated, self-administered, cross-sectional questionnaire used in Italy, and revised based on a literature review. The final questionnaire was pre-tested prior to data collection, and covered a broad range of domains assessing the KAP of participants, including but not limited to common uses of antibiotics and prescription practices, among other factors. Face validity was established using a panel of experts who thoroughly reviewed the questionnaire and concluded that it measures the traits of interest. Additionally, we field-tested the questionnaire to understand how well the instrument is able to collect the intended information. We also conducted a Cronbach’s alpha test to assess internal reliability: the alpha score of 0.75 indicated moderate reliability of the questionnaire.

### Recruitment and sampling

The study team adopted a convenience sampling methodology to recruit participants for the KAP survey due to resource limitations. Based on a study describing antibiotic prescription practices for acute, uncomplicated respiratory tract infections [23], we assumed a prevalence of irrational antibiotic usage (overuse and inappropriate choice of antibiotics for treatment of viral infections) among health care personnel of 50% with alpha = 5%, design effect of 1%, and 5% acceptable margin of error. The minimum sample size was calculated at 384. The sampling frame was subsequently stratified among the four groups of healthcare providers: 96 allopathic doctors, 96 informal healthcare providers, 96 nurses, and 96 pharmacy shopkeepers. It must be noted that while the intent was to assess the KAP of pharmacists, we could not ascertain whether participants were licensed pharmacists, as many shops dispensing antibiotics were not licensed pharmacies. We have included all participants who were interviewed at pharmacies as “pharmacy shopkeepers” in the study. With this sample size, the power of the study to detect a 20% difference in composite scores between allopathic doctors, informal healthcare providers, nurses, and pharmacy shopkeepers was 82% based on a normal approximation with continuity correction. 60% of doctors had a low composite KAP score (<80) in comparison to 80% of all participants in the other occupation groups. We considered a two-sided confidence interval of 95%, with a sample size of 96 in each occupational group.

The primary health system in India is organized into primary, secondary, and tertiary levels, with subcentres (SCs) and primary health centres (PHCs) constituting the first point of entry, community health centres and smaller sub-district hospitals constituting secondary care, and medical colleges and district hospitals constituting tertiary care within the government system. The study team systematically started at the primary health centre and subcentre levels to target informal healthcare providers and allopathic practitioners working at the grassroots level, and worked their way upwards to block primary health centres (BPHCs) and Asansol District Hospital (ADH), the main secondary care referral centre in the district. In total, all 8 administrative blocks of Paschim Bardhaman district were covered. BPHCs and associated PHCs were sampled from Khandra, Kelejora, Laudoha, Akalpur, Bahadurpur, Panagarh, Raniganj and Pithaikeary.

### Data collection and analysis

Data collection took place over a period of approximately 8 weeks from December to February following an initial one month period of planning and field mapping. We continuously approached participants to attain our target sample size of 384 respondents. In total, we approached 494 people to participate in the survey, and 110 declined to participate, yielding a response rate of 77.7%. The most commonly cited reason for declining was a lack of interest in the study. All study participants were assured for confidentiality and informed that the data would be de-identified at entry. Participants were only identified by an ID number in the database and participant names only appeared in the consent forms. Survey responses for all 384 participants were collated in Excel and data was analyzed in SPSS. Each Likert scale question received a maximum of 5 points for the most accurate answer, and 1 point for the least accurate answer. As such, there was a maximum possible score of 32, 45, and 20 in the knowledge, attitudes, and practice sections respectively, and a maximum total composite score of 97. Our team created an equivalent, composite KAP score for each respondent in the survey, which was subsequently compared between providers. We then performed a multivariate logistic regression analysis to estimate the odds of having a low composite score (<80) associated with occupation (comparing the referent category of allopathic doctors with all other study respondents), adjusted for age (included as a continuous variable) and gender (male or female). While gender was significantly associated with low score in the univariable analysis, age showed no significant relationship. However, we still chose to include age in the final model. The level of significance was set at a p-value<0.05, and a 95% confidence interval was calculated for all odds ratio calculations. Apart from checking the level of skewedness and kurtosis, the normality of variables was also assessed using a test of normality (Shapiro-Wilk test) in SPSS.

## Results

### Demographic characteristics

Over 90% of the participants were between 26 and 60 years of age (Table 1). Amongst pharmacy shopkeepers, 9.4% (n = 9) were between the ages of 18–25 years; our field observations suggested that several of these workers were not actually licensed pharmacists, but rather apprentices or relatives of small business owners. They were not asked to provide documentary evidence of their qualifications. The mean age of all participants in the study was observed at 41.7 years (± 10.9 years). Of the 19 variables related to KAP questions, only 4 had 100% complete data. The remaining 15 variables had a median (IQR) of 3 (2–6) records missing in the database out of 384 records for each variable.

**Table 1:** Demographic characteristics of health provider sub-groups.

	Allopathic* (N = 96) N (%)	Informal providers* (N = 96) N (%)	Nurses* (N = 96) N (%)	Pharmacy shopkeepers* (N = 96) N (%)	Total (N = 384) N (%)
**Age group (years)**					
18–25	0 (0)	1 (1.0)	3 (3.1)	9 (9.4)	13 (3.4)
26–35	36 (37.5)	14 (14.6)	32 (33.3)	23 (24.0)	105 (27.3)
36–45	19 (19.8)	41 (42.7)	38 (39.6)	37 (38.5)	135 (35.2)
45–60	35 (36.5)	33 (34.4)	23 (24.0)	20 (20.8)	111 (28.9)
>60	6 (6.2)	7 (7.3)	0 (0)	7 (7.3)	20 (5.2)
**Mean + SD age (years)**					
	42.5 ± 11.5	44.2 ± 10.1	39.9 ± 10.2	40.2 ± 11.2	41.7± 10.9
**Gender**					
Male	76 (79.2)	95 (99)	0 (0)	93 (96.9)	264 (68.8)
Female	20 (20.8)	1 (1)	96 (100)	3 (3.1)	120 (31.3)
**Work setting**					
PHC	31 (32.3)	0 (0)	61 (63.5)	11 (11.5)	103 (26.8)
District hospital	61 (63.5)	2 (2.1)	35 (36.5)	0 (0)	98 (25.5)
Private hospital	0 (0)	1 (1.0)	0 (0)	0 (0)	1 (0.3)
Private clinic	3 (3.1)	90 (93.8)	0 (0)	2 (2.1)	95 (24.7)
Pharmacy	0 (0)	3 (3.1)	0 (0)	81 (84.4)	84 (21.9)
Others	1 (1.0)	0 (0)	0 (0)	2 (2.1)	3 (0.8)
**Work experience (months)**					
1–11	13 (13.5)	0 (0)	3 (3.1)	4 (4.2)	20 (5.2)
12–60	29 (30.2)	12 (12.5)	29 (30.2)	21 (21.9)	91 (23.7)
61–120	17 (17.7)	9 (9.4)	23 (24.0)	17 (17.7)	66 (17.2)
>120	37 (38.5)	75 (78.1)	41 (42.7)	54 (56.3)	207 (53.9)

*All four prescriber groups had statistically significant differences for each demographic characteristic (chi square: p-value < 0.001)

Overall, the vast majority of participants (68.8%) were male: the proportions of informal health providers and pharmacy shopkeepers were heavily skewed towards men, whereas nurses were entirely female. While doctors and nurses were overwhelmingly drawn from the government setup (either Asansol District Hospital or PHCs), almost all of the informal health providers (93.8%) were interviewed at their private clinic and 84.4% of pharmacy shopkeepers were interviewed at their pharmacy shop. Furthermore, just over half of participants (53.9%) had over 10 years of work experience, a finding that was particularly notable among informal health providers (78.1%) who claimed to rely heavily on experience for decision making in their professional work.

### Dissonance between knowledge and practice

The 38-point survey questionnaire provided a holistic overview of the knowledge, attitudes, and practices related to antibiotic use. The most pronounced finding was the dissonance between knowledge and practices, wherein participants could correctly identify appropriate uses of antibiotics, and yet fail to apply these findings in practice.

As a starting point, all doctors claimed to prescribe antibiotics in their practice. Several patients were given at least one antibiotic for a multitude of reasons: some claimed it was due to patient demands, while others claimed to use antibiotics as a precautionary measure to prevent against secondary infections. Nearly all (95.7%) informal health providers and half of pharmacy shopkeepers who were interviewed reported prescribing antibiotics at their workplace.

**Fig 1:**
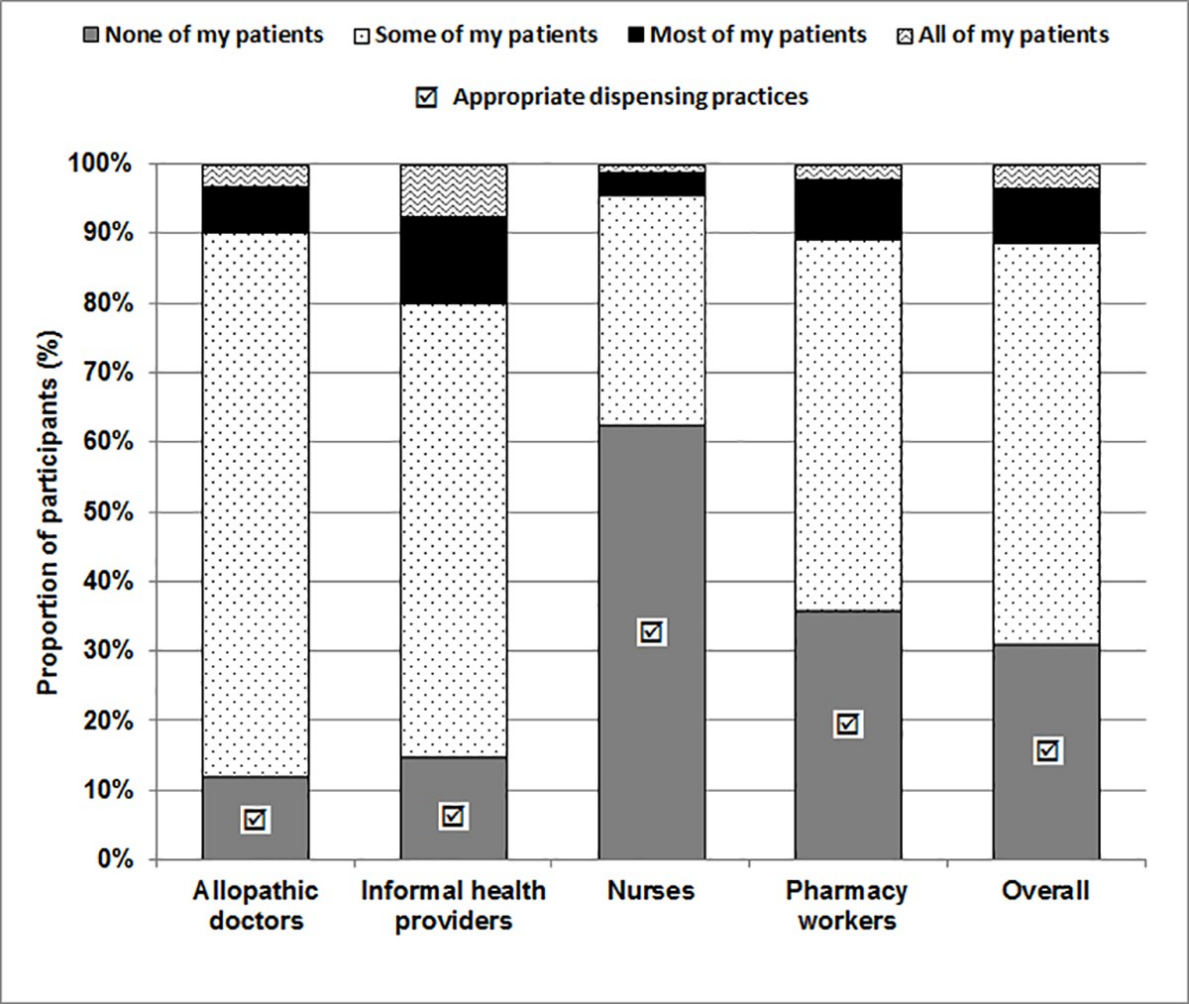
Proportion of healthcare providers dispensing antibiotics for a cold or sore throat.

Similarly, when asked how frequently health providers prescribe or provide an antibiotic for symptoms of common cold or sore throat (Fig 1), over 88% of allopathic doctors and 85% of informal health providers responded by saying they did this for at least some of their patients. We considered “none of my patients” as the only acceptable and appropriate response for all providers. Clinical guidelines indicate that antibiotics are ineffective for symptoms of the common cold; as a result, any antibiotic prescriptions for these symptoms would be considered inappropriate [24].

Furthermore, 7.4% of informal health providers claimed they prescribed or provided an antibiotic to all of their patients for symptoms of a common cold or sore throat.

### Attitudes are very similar across respondent groups

Interestingly, 95.9% of informal health providers, 98.9% of nursing staff, and 94.8% of pharmacy shopkeepers claimed that knowledge of antibiotics was important to them in their role as a health provider, even though none of these groups are legally permitted to prescribe antibiotics independently. Where knowledge of antibiotics was highly valued, participants stated that their most utilized sources of information were medical textbooks, pharmaceutical company representatives (PCRs), and allopathic doctors (in the case of informal health providers).

### Knowledge differences between provider groups

**Fig 2:**
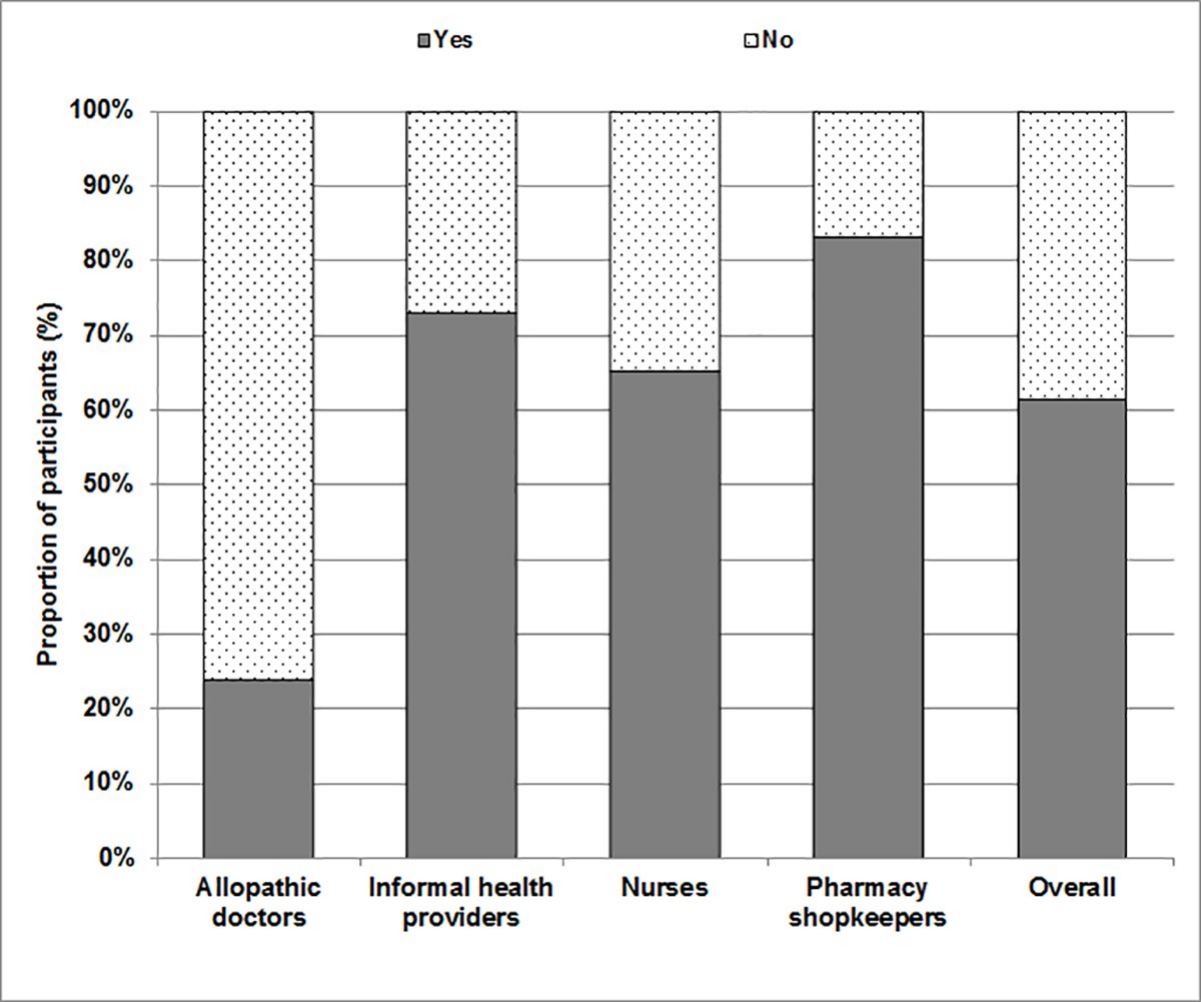
Proportion of healthcare providers expressing agreement with the statement that antibiotics are useful for viral infections.

All four groups of participants were asked whether antibiotics were useful for viral infections and whether antibiotics were indicated to reduce the symptoms of pain and inflammation as part of the knowledge assessment. As Fig 2 below indicates, the levels of knowledge of allopathic doctors contrasted strongly with the other three groups as expected. 76.1% of allopathic doctors either strongly disagreed or disagreed with the statement that antibiotics were useful for viral infections, and these were combined as “no” responses in Fig 2. Neutral answers were included as “yes” responses, as a neutral response to such a question does not concur with current medical guidelines [24]. A few senior doctors who responded incorrectly later qualified their answers by suggesting that a lack of follow-up opportunities and the likelihood of developing a secondary infection occasionally required the use of antibiotics even during viral infections. Similar sentiments were expressed by allopathic doctors when they were asked whether antibiotics were indicated to alleviate the symptoms of pain and inflammation: a few doctors explicitly stated that inflammation can be caused by a bacterial infection, which would necessitate the use of antibiotics even in the absence of confirmation from laboratory testing.

### Knowledge of contraindications of antibiotics during pregnancy

Most allopathic doctors confidently completed the survey questionnaire without much hesitation. However, the most challenging question for allopathic doctors inquired which antibiotic was contraindicated in pregnancy, with three possible single-select options: Amoxicillin, Ciprofloxacin, and Gentamicin. The U.S. Food & Drug Administration classifies Gentamicin as a Category D drug, indicating that there is positive evidence of fetal risk in humans [25]. By contrast, Ciprofloxacin is classified as a Category C drug, which indicates that there are no adequate and controlled studies in humans despite animal reproduction studies indicating adverse effects. The correct answer (Gentamicin) was selected by a mere 43.8% of doctors, while another 43.8% of doctors incorrectly identified Ciprofloxacin. Only 17.7% of pharmacy shopkeepers correctly identified Gentamicin, while 54.2% claimed they did not know. Informal health providers and nurses similarly had little idea about Gentamicin and its links to potential birth defects, even though we frequently observed IHPs providing Gentamicin during field observations.

### Awareness regarding the importance of a full course of antibiotics

**Fig 3:**
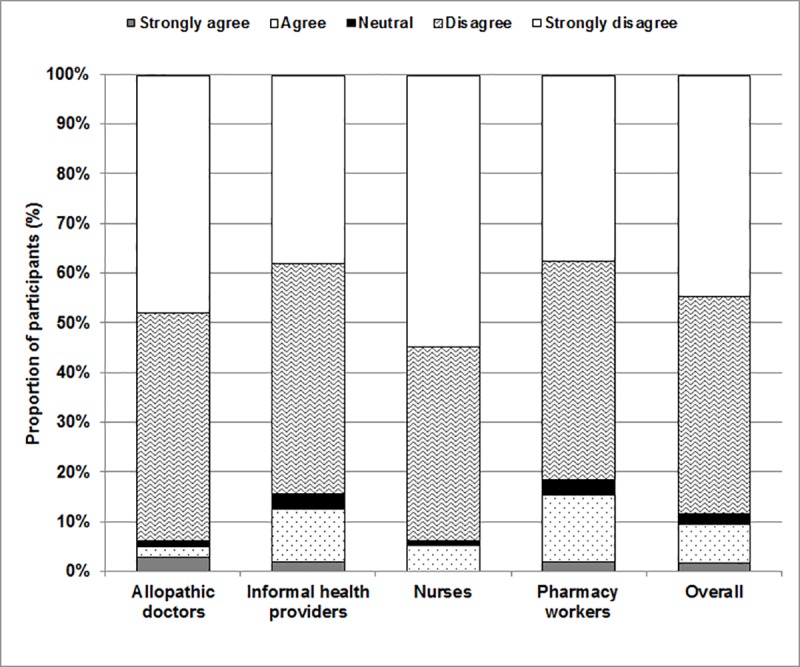
Proportion of healthcare providers who find it appropriate to stop taking antibiotics prior to the completion of the full course.

One of the more interesting questions in our study was an assessment of the respondent’s awareness around the full course of antibiotics. As Fig 3 below demonstrates, an overwhelming proportion of all respondents either strongly disagreed or disagreed with the practice of stopping a full course before it was complete. However, this also reflected a sharp dissonance between knowledge and practice, as we found that all four groups of respondents frequently disbursed shorter, 3-day courses of antibiotics rather than a full course.

### Composite KAP scores

Our team conducted a more detailed analysis of the knowledge, attitudes, and practices by creating a composite KAP score for each respondent in the survey, which has been presented as average percentage scores for ease of comparison below in Table 2.

**Table 2:** Average percent scores for knowledge, attitudes, and practices by occupation.

Occupation	Knowledge Mean % (SD)	Attitudes Mean % (SD)	Practice Mean % (SD)	Composite KAP score Mean % (SD)
Allopathic doctor	77.3 (12.7)	87.3 (7.7)	67.6 (11.1)	79.9 (6.9)*
Informal health providers	56.9 (11.5)	80.0 (9.6)	71.2 (11.6)	70.6 (7.8)*
Nurses	63.4 (11.2)	79.9 (9.2)	75.5 (12.6)	73.5 (7.2)*
Pharmacy shopkeepers	57.8 (9.7)	79.3 (9.6)	72.1 (12.8)	70.7 (7.0)*

*Statistically significant difference in composite scores between occupational groups (p<0.001)

One-way ANOVA testing determined that there was a statistically significant difference in average scores of knowledge, attitude and practice questions between the four occupational groups. As evidenced in Table 2, allopathic doctors scored highest in questions assessing knowledge (77.3%) and attitudes (87.3%), and yet, this knowledge did not translate into practice. Allopathic doctors performed most poorly (67.6%) when asked about their practices with respect to antibiotic use and prescriptions.

We also conducted a multivariate logistic regression analysis, adjusting for age and gender, and found that the odds of having a low composite score in non-allopathic practitioners was 10.4 (95% CI 5.4, 20.0) times greater than allopathic doctors (p<0.001). Thus, our study found that non-allopathic practitioners were significantly more likely to perform poorly on the survey.

## Discussion

Our study represents one of the few attempts in the literature to assess the knowledge, attitudes, and practices of all providers interacting with antibiotics in the Indian context, including allopathic doctors, nurses, pharmacy shopkeepers, and informal health providers. While doctors outperformed other formal and informal healthcare providers in the knowledge and attitudes components of the survey, their comparatively poor performance in the practice section of the survey is concerning. This may be due to the fact that many doctors across India prescribe antibiotics in PHC settings as a precautionary measure to compensate for diagnostic uncertainty due to lack of availability of point-of-care diagnostic tests, poor infection control, and inadequate sanitation practices [26]. Additionally, informal practitioners, nurses, and pharmacy shopkeepers are not legally entitled to prescribe antibiotics, which may have prompted them to answer less candidly on practice-related questions in comparison to doctors.

Other studies [11–13, 18–20] have focused predominantly on the KAP of medical students or clinicians in tertiary care hospitals, which address a limited part of the antibiotic dispensing pathway and leave out the crucial role played by informal providers. These studies generally report poor practices even among medical students and allopathic practitioners: for instance, Khan et al. found that only 77.3% (n = 75) of respondents were aware that bacteria were not responsible for causing colds and flu [20]. Another study by Gautham et al. compares the knowledge, practices and relationships of informal providers in two districts in Northern and Southern India [27], and finds their role has evolved differently in the two disparate market settings. The study documents not just wide variations in knowledge, but also interesting and mutually beneficial referral links with private doctors.

While the presence of informal providers is particularly salient in India, such providers can be found in every health system, according to the WHO [28]. A systematic review conducted by Sudhinaraset et al. finds that people across developing countries use informal providers due to convenience, affordability, and social and cultural preferences; many providers across countries such as Vietnam and Bangladesh reported poor adherence to national guidelines with respect to antibiotic use [29]. In developed economies such as the United States, antibiotics are only prescribed by graduate physicians, but similar problems persist with respect to inappropriate overuse of antibiotics: recent studies estimated that at least 30% of all antibiotic prescriptions in outpatient settings in the United States are considered inappropriate, and 50% of all antibiotic prescriptions for respiratory tract infections are inappropriate [30–31]. The authors found that inappropriate prescription practices were linked to a multitude of factors, including clinical factors, demographic characteristics of patients, severity of illness, previous infection history, compromised immune response, geographic region, among others [30]. Multi-center studies conducted across the United States, Scotland, Switzerland, Sweden, Slovenia, Spain, France, and England examined knowledge, attitudes, and practices related to antibiotic use among medical students and found that the vast majority wished to acquire more knowledge about choosing appropriate antibiotic treatment [32–34]. Moreover, many relied on Wikipedia more than formal peer-reviewed sources or textbooks for guidance on antimicrobial use [33].

In our context, many providers relied on pharmaceutical company representatives as a source of information regarding new antibiotics and associated uses. This may stem from an inadequate focus on antibiotics and drivers of antibiotic resistance in the medical curriculum. Our findings further indicate that there is poor knowledge and awareness of antibiotic uses and functions among informal providers and a strong dissonance between knowledge and practices among formal healthcare providers. This is supported by other studies as well: Das et al. have demonstrated significant quality gaps between private and public providers of primary health care in other Indian states, noting incorrect medical diagnoses, incorrect treatments, and inconsistent adherence to clinical checklists [35]. Similarly, Scaioli et al used the original questionnaire adapted in our study on a convenience sample of students from medical, dental, nursing, and other health professions in Italy and found that health professionals do not practice what they know: in other words, high levels of knowledge do not translate into appropriate attitudes and practices with respect to antibiotic use [36]. We found that allopathic doctors treated common illnesses such as cold, cough, fever, and watery loose stools with antibiotics without clinical indication, largely due to risk aversion in the context of diagnostic uncertainty and lack of robust follow-up.

As for informal providers, Bloom et al. note in their study on informal providers in health markets in Nigeria and Bangladesh that the behavior of informal providers is governed predominantly by the institutional context in which they operate and the emergence of such informal markets represents broader failures in the public health delivery system to meet the needs of the impoverished [37]. This may explain the high rates of antibiotic use among informal providers in our context, since many areas of the district are inadequately covered by existing public facilities. Previous studies have advocated for re-training and “formalizing” the informal health providers by empowering them to practice allopathic medicine as “social physicians,” given concerns around equity and access to care for rural populations [38, 39]. However, the evidence on training programs is uncertain: Das et al. recently examined the impact of training informal healthcare providers in West Bengal and found that while the training increased correct case management rates, it did not reduce unnecessary antibiotic use [40]. Rather than focusing solely on training, future efforts must work to strengthen the existing public health infrastructure.

The Government of India has also embraced this idea and announced a Bridge Program in Community Health for Nurses and Ayurveda practitioners under the flagship National Health Mission in order to enhance the capacity of mid-level care providers [41]. Initiatives like this can be scaled up to improve the capacity of the existing public health infrastructure to meet patient demands. We argue that these kinds of initiatives provide a unique opportunity to enhance awareness around antibiotic resistance while ensuring equitable access to antibiotics when required. Balancing the demands between access and overuse of antibiotics is the big challenge for policy makers at present.

In order to better tackle the growing threat of resistance, India also adopted the National Action Plan on Antimicrobial Resistance (2017–21) in April [42]. The objectives include reducing infections, enhancing awareness, strengthening surveillance, improving rational use, promoting research and supporting neighboring countries in the collective fight against infectious diseases. However, the National Plan does not account for the role of PCRs and informal providers with respect to antibiotic use. Trainings should be initiated for PCRs who have been largely ignored in the scientific literature around antibiotic resistance. In Paschim Bardhaman district, the vast majority of participants surveyed indicated that they relied on pharmaceutical company representatives as a source of medical knowledge.

Future efforts to curb antibiotic resistance should involve advocacy with pharmaceutical firms in order to tap into the strong, pre-existing networks of PCRs and reorient their efforts to promote a more responsible message around antibiotics; regulation may also be useful in addressing the issue. These efforts to involve PCRs and informal health providers can form a critical community-level component of antibiotic stewardship programs moving forward, in addition to emphasizing antibiotic resistance in the curriculum for continued medical education, increasing access to cost-effect point-of-care diagnostics to aid doctors in decision making, increasing the use of prescription audits in primary care settings, and enforcing the legal framework around over-the-counter use of antibiotics. Current initiatives to tackle ABR in Asia aim to set up surveillance systems, regulate the sale of antibiotics, and introduce national guidelines for antibiotic use, but fail to take into account patient perceptions and expectations from health providers as a major driver for ABR. Any effort to tackle antibiotic resistance must also include patient education and counseling, as patient demands are a major driver for overuse of antibiotics among formal and informal health providers alike. Several recent studies demonstrate that the Internet and social media, in particular, can be an effective resource for disseminating high quality health information to improve antibiotic stewardship in the community [43–44]. Future interventions must consider social media within their communication strategy to promote appropriate use for antibiotic-related information seeking in the general population.

### Limitations

This study had several limitations due to resource constraints. Firstly, given the use of convenience sampling in the survey design, our findings must be interpreted with caution when generalized to Paschim Bardhaman district despite the sample size of 384 participants. The use of convenience sampling limits the generalizability of the results at the study population level and may introduce the potential for selection bias in the study. Secondly, the overall population being studied may not be representative: for instance, we were not able to ascertain whether pharmacy shopkeepers were licensed or operating informally; thus, the “pharmacy shopkeepers” sample may not be representative of the overall population of pharmacists in the region. Finally, self-reported data always comes with limitations. We were unable to cross-check survey responses with actual prescription practices, and social desirability bias may have played a role in undermining the credibility of our results. This may explain why doctors performed more poorly than informal providers in the “practices” section of the validated questionnaire. Given the vastly limited literature from this region of India, we believe the study adds value in understanding the scope of the problem, but we recommend that future studies should be undertaken with a robust study design in mind.

## Supporting information

S1 Appendix Survey reporting form. (XLS)

S2 Appendix English questionnaire. (PDF)

S3 Appendix Bengali questionnaire. (PDF)

## Abbreviations

ABR: Antibiotic Resistance
ADH: Asansol District Hospital
BPHC: Block Primary Health Centre
CMOH: Chief Medical Officer of Health
DNB: Diplomate of National Board
ERB: Ethical Review Board
ICF: Informed Consent Form
ICMR: Indian Council of Medical Research
IEC: Information Education Communication
IHP: Informal Health Provider
KAP: Knowledge, Attitudes, Practices
KMC: Kasturba Medical College
MOH: Ministry of Health
MRSA: Methicillin Resistant Staphylococcus Aureus
MSF: Medecins Sans Frontieres
OPD: Out-patient Department
OTC: Over-the-counter
PCR: Pharmaceutical Company Representatives
PHC: Primary Health Centre
SC: Sub-Centre
WHO: World Health Organization
