# Supplementary material for: Knowledge, attitudes, and practices related to antibiotic use in Paschim Bardhaman District: A survey of healthcare providers in West Bengal, India
Source: PLoS One. 2019 May 31;14(5):e0217818. doi: 10.1371/journal.pone.0217818 (PMC6544287; doi:10.1371/journal.pone.0217818)
Supplement: S2 Appendix — (PDF) [file pone.0217818.s002.pdf]

ID #: ____________________

QUESTIONNAIRE FOR KNOWLEDGE, ATTITUDES, AND PRACTICES REGARDING THE USE OF ANTIBIOTIC

1. Age: ____________________

2. Gender (*tick one*): □ Male □ Female

3. Block: ______________________________

4. Highest education level: _________________________________

5. Occupation:
   - □ Allopathic (qualified/trained, MBBS+) doctor
   - □ AYUSH doctor
   - □ Registered medical practitioner (RMP)
   - □ Untrained medical provider (UMP)
   - □ Pharmacist
   - □ Other (*please specify:* _____________________)

6. Work setting:
   - □ Primary health centre (*name*: __________________________)
   - □ District hospital (*name*: ______________________________)
   - □ Private hospital (*name*: _______________________________)
   - □ Private clinic (*name*: _________________________________)
   - □ Pharmacy (*name*: ___________________________________)
   - □ Other (*please specify:* _________________________________)

7. How long have you worked in your present job? ______ years
   ______ months

8. Average number of patients seen during home visits: _____________
9. Average number of patients seen at your clinic/stall: ______________
10. Average fees charged (*check all that apply and please specify amount*):
    - □ Consultation fees: ____________________
    - □ Medicines: _________________________
    - □ Other (*please specify type of fee and amount*): ________________________
11. Most patients coming to you are:
    - □ Illiterate
    - □ Basic or primary education
    - □ Secondary education and above
12. Most patients coming to you are:
    - □ Daily wage earners
    - □ Landed agriculturalists (farmers with land)
    - □ Salaried workers
    - □ Other informal occupation (*please specify:* __________________________)

13. Top three diseases usually seen:
    1) ___________________
    2) ___________________
    3) ___________________
14. Knowledge of antibiotics is important to me in my role as a health provider.
    □ Strongly agree
    □ Agree
    □ Neutral
    □ Disagree
    □ Strongly disagree
15. Usual source of information on antibiotics or other drugs:
    □ Literature provided by medical representatives
    □ Information obtained from other physicians
    □ Internet or other online platform (*please specify which platforms or websites):*
    ____
    □ Some other platform (*please specify):*
    ____
16. Do you think you have enough sources of information about antibiotics when you need it?
    □ Yes (*please specify which sources you use*):
    ____
    □ No (*please specify which sources you would like to have available*):
    ____
17. Do you know of any guidelines related to antibiotic use?
    □ Yes (*please name the guidelines*):
    ____
    □ No
18. Do you think it is difficult to select the correct antibiotic for a particular illness?
    □ Yes (*please explain*):
    ____
    □ No (*please explain*):
    ____
19. Antibiotics are useful for bacterial infections (i.e. typhoid)
    □ Strongly agree
    □ Agree
    □ Neutral
    □ Disagree
    □ Strongly disagree
20. Antibiotics are useful for viral infections (i.e. flu)
    □ Strongly agree
    □ Agree
    □ Neutral
    □ Disagree
    □ Strongly disagree
21. Antibiotics are indicated to reduce the symptoms of pain and inflammation
    □ Strongly agree
    □ Agree
    □ Neutral
    □ Disagree
    □ Strongly disagree

22. Antibiotic resistance is a phenomenon for which a bacterium loses its sensitivity to an antibiotic
    - □ Strongly agree
    - □ Agree
    - □ Neutral
    - □ Disagree
    - □ Strongly disagree
23. A 40 year-old woman comes to you complaining of 4 days of watery loose stools with 1-2 episodes of vomiting. No history of fever. Which antibiotic will you recommend?
    - □ Ciprofloxacin
    - □ Metronidazole
    - □ Trimethoprim-sulfamethoxazole
    - □ No need of antibiotic use. Oral rehydration only.
24. Which <u>one</u> of the following antibiotics is contraindicated in pregnancy?
    - □ Amoxicillin
    - □ Ciprofloxacin
    - □ Gentamicin
25. Antibiotics are overused in the community in India
    - □ Strongly agree
    - □ Agree
    - □ Neutral
    - □ Disagree
    - □ Strongly disagree
26. Patients' demands for antibiotics contribute to overuse
    - □ Strongly agree
    - □ Agree
    - □ Neutral
    - □ Disagree
    - □ Strongly disagree
27. General knowledge on antibiotic resistance should be considered when antibiotics are prescribed to an individual patient
    - □ Strongly agree
    - □ Agree
    - □ Neutral
    - □ Disagree
    - □ Strongly disagree
28. I believe that prescribing antibiotics is harmful to patients if not indicated.
    - □ Strongly agree
    - □ Agree
    - □ Neutral
    - □ Disagree
    - □ Strongly disagree
29. I would like access to educational programs on antibiotic prescription.
    - □ Strongly agree
    - □ Agree
    - □ Neutral
    - □ Disagree
    - □ Strongly disagree

30. I can do something to combat antibiotic resistance in my practice.
    - □ Strongly agree
    - □ Agree
    - □ Neutral
    - □ Disagree
    - □ Strongly disagree
31. Antibiotic resistance is a problem worldwide
    - □ Strongly agree
    - □ Agree
    - □ Neutral
    - □ Disagree
    - □ Strongly disagree
32. Antibiotic resistance is a problem in my daily practice
    - □ Strongly agree
    - □ Agree
    - □ Neutral
    - □ Disagree
    - □ Strongly disagree
33. How frequently do you review your decision to prescribe antibiotics with a colleague?
    - □ Never
    - □ Sometimes
    - □ Most of the time
    - □ Always
34. How frequently do you prescribe antibiotics in your practice?
    - □ None of my patients
    - □ Some of my patients
    - □ Most of my patients
    - □ All of my patients
35. How frequently do you prescribe or provide an antibiotic for a cold or sore throat?
    - □ None of my patients
    - □ Some of my patients
    - □ Most of my patients
    - □ All of my patients
36. A patient has been prescribed a full course of antibiotics. She stopped it after 3-4 doses as she had started feeling better. Do you find this appropriate?
    - □ Strongly agree
    - □ Agree
    - □ Neutral
    - □ Disagree
    - □ Strongly disagree
37. How frequently do you explain the proper usage of antibiotics to patients?
    - □ Never
    - □ Sometimes
    - □ Most of the time
    - □ Always
38. What proportion of patients coming to your clinic/pharmacy seek antibiotics?
    - □ None of my patients
    - □ Some of my patients
    - □ Most of my patients
    - □ All of my pa3ients
