# Supplementary material for: Knowledge, attitudes, and practices related to antibiotic use in Paschim Bardhaman District: A survey of healthcare providers in West Bengal, India
Source: PLoS One. 2019 May 31;14(5):e0217818. doi: 10.1371/journal.pone.0217818 (PMC6544287; doi:10.1371/journal.pone.0217818)
Supplement: S3 Appendix — (PDF) [file pone.0217818.s003.pdf]

ID: ....

অ্যান্টিবায়োটিক ব্যাবহার সম্বন্ধে জ্ঞান, মনোভাব এবং অভ্যাস সম্পর্কিত প্রশ্নাবলী

১. বয়স: ....

২. লিঙ্গ: (একটিতে টিক দিন) ☐ স্ত্রী ☐ পুরুষ

৩. ব্লক: ....

৪. উচ্চতম শিক্ষাগত যোগ্যতা: ....

নিম্নলিখিত প্রশ্নগুলির জন্য দয়া করে যে কোনো একটি উত্তর নির্বাচন করুন:

৫. পেশা:

☐ অ্যালোপ্যাথি (যোগ্যতাসম্পন্ন/প্রশিক্ষিত, MBBS+) ডাক্তার
☐ আয়ুষ ডাক্তার
☐ রেজিস্টার্ড মেডিক্যাল প্র্যাকটিশনার (RMP)
☐ আনট্রেন্ড মেডিক্যাল প্রোভাইডার (UMP)
☐ ফার্মাসিস্ট
☐ অন্যান্য (দয়া করে উল্লেখ করুন ....)

৬. কর্মক্ষেত্র:

☐ প্রাথমিক স্বাস্থ্যকেন্দ্র (নাম:...)
☐ জেলা হাসপাতাল (নাম:...)
☐ বেসরকারি হাসপাতাল (নাম:...)
☐ প্রাইভেট ক্লিনিক (নাম:...)
☐ ফার্মাসি (নাম:...)
☐ অন্যান্য (দয়া করে উল্লেখ করুন ....)

৭. আপনার বর্তমান চাকরিতে আপনি কতদিন কাজ করছেন? .... বছর .... মাস

৮. গড়ে কতজন রোগীকে আপনি বাড়ীতে গিয়ে দেখেন: ....

৯. গড়ে কতজন রোগীকে আপনি ক্লিনিকে অথবা দোকানে দেখেন: ....

১০. গড়ে আপনি কত ফি নেন (যেগুলো প্রযোজ্য সেগুলো টিক দিন এবং নির্দিষ্ট মূল্য লিখুন):

☐ কন্সালটেশন ফি:..
☐ ঔষুধ:...
☐ অন্যান্য (দয়া করে কি ধরনের ফি এবং পারিমান উল্লেখ করুন): ....

১১. আপনার কাছে আসা রোগীরা বেশিরভাগই :

☐ অশিক্ষিত
☐ প্রাথমিক শিক্ষিত
☐ মাধ্যমিক বা আরও উচ্চতর শিক্ষিত

১২. আপনার কাছে যে রোগীরা আসেন তাদের বেশিরভাগই :

☐ দিনমজুর
☐ ভূমি-কৃষক (যার নিজের জমি আছে)
☐ বেতনভোগী কর্মচারী
☐ অন্যান্য অপ্রথাগত জীবিকা (দয়া করে উল্লেখ করুন ....)

১৩. সর্বাধিক তিনটি রোগ যেগুলো দেখা যায় :

১) ………………………………………….
২) ………………………………………….
৩) ………………………………………….

১৪. একজন স্বাস্থ্যকর্মী হিসাবে অ্যান্টিবায়োটিক সম্পর্কে জ্ঞান থাকা আমার কাছে গুরুত্বপূর্ণ

☐ দৃঢ়ভাবে একমত
☐ একমত
☐ নিরপেক্ষ
☐ অসম্মত
☐ দৃঢ়ভাবে অসম্মত

১৫. অ্যান্টিবায়োটিক বা অন্যান্য ওষুধ ব্যাবহারের ক্ষেত্রে তথ্যের প্রচলিত উৎস হল:

☐ মেডিকেল রিপ্রেজেন্টেটিভ দের দেওয়া তথ্য
☐ অন্যান্য ডাক্তারদের থেকে পাওয়া তথ্য
☐ ইন্টারনেট বা অন্যান্য অনলাইন মাধ্যম থেকে (দয়া করে অনলাইন মাধ্যমটি বা ওয়েবসাইট উল্লেখ করুন) ……………………………………………………………………………………………………
☐ অন্যান্য কোনো মাধ্যম (দয়া করে উল্লেখ করুন ……………………………………………………)

১৬. আপনি কী মনে করেন যে, যখন প্রয়োজনমতো আপনি অ্যান্টিবায়োটিক দেন তখন আপনার কাছে অ্যান্টিবায়োটিক সম্পর্কে যথেষ্ট তথ্য আছে?

☐ হ্যাঁ (আপনার তথ্যের উৎস দয়া করে উল্লেখ করুন) : ……………………………………………………………………………..

☐ না (কোন্ উৎস থেকে তথ্য পেতে চাইবেন তা দয়া করে উল্লেখ করুন) : ……………………………………………………………………………

১৭. আপনি কি অ্যান্টিবায়োটিক ব্যবহার সম্পর্কিত কোনও গাইডলাইন জানেন?

☐ হ্যাঁ (গাইডলাইনের নাম দয়া করে উল্লেখ করুন) : ……………………………………………………………………………..

☐ না

১৮. আপনি কি একটি বিশেষ অসুস্থতার জন্য সঠিক অ্যান্টিবায়োটিক নির্বাচন করা কঠিন বলে মনে করেন?

☐ হ্যাঁ (দয়া করে ব্যাখ্যা করুন) : ……………………………………………………………………………..

☐ না (দয়া করে ব্যাখ্যা করুন) : ……………………………………………………………………………..

১৯. অ্যান্টিবায়োটিকগুলি ব্যাকটেরিয়া সংক্রমণের ক্ষেত্রে উপযোগী (যেমন-টাইফয়েড).

☐ দৃঢ়ভাবে একমত
☐ একমত
☐ নিরপেক্ষ
☐ অসম্মত
☐ দৃঢ়ভাবে অসম্মত

২০. অ্যান্টিবায়োটিকগুলি ভাইরাস ঘটিত সংক্রমণের ক্ষেত্রে উপযোগী (যেমন- জ্বর)

- ☐ দৃঢ়ভাবে একমত
- ☐ একমত
- ☐ নিরপেক্ষ
- ☐ অসম্মত
- ☐ দৃঢ়ভাবে অসম্মত

২১. ব্যথা এবং প্রদাহের/ক্ষতর লক্ষণ কমাতে অ্যান্টিবায়োটিকের প্রয়োজন হয়

- ☐ দৃঢ়ভাবে একমত
- ☐ একমত
- ☐ নিরপেক্ষ
- ☐ অসম্মত
- ☐ দৃঢ়ভাবে অসম্মত

২২. অ্যান্টিবায়োটিক প্রতিরোধ এমন একটি অবস্হা যেক্ষেত্রে একটি ব্যাকটেরিয়া, একটি অ্যান্টিবায়োটিক দ্বারা তার কার্যক্ষমতা হারায়

- ☐ দৃঢ়ভাবে একমত
- ☐ একমত
- ☐ নিরপেক্ষ
- ☐ অসম্মত
- ☐ দৃঢ়ভাবে অসম্মত

২৩. একটি 40 বছর বয়সী মহিলা আপনার কাছে এসে জানালেন 4 দিন ধরে জলের মতো পাতলা পায়খানা এবং 1-2 বার বমি হয়েছে। জ্বর আসেনি। কোন্ অ্যান্টিবায়োটিক আপনি নেবার পরামর্শ দেবেন?

- ☐ Ciprofloxacin
- ☐ Metronidazole
- ☐ Trimethoprim-sulfamethoxazole
- ☐ অ্যান্টিবায়োটিক-র প্রয়োজন নেই, শুধু ORS দিলেই হবে।

২৪. গর্ভাবস্থায় নিম্নলিখিত অ্যান্টিবায়োটিকগুলির মধ্যে কোনটি দেওয়া উচিত না?

- ☐ Amoxicillin
- ☐ Ciprofloxacin
- ☐ Gentamicin

২৫. ভারতবর্ষে সাধারন মানুষ প্রয়োজনের অতিরিক্ত অ্যান্টিবায়োটিক ব্যবহার করেন

- ☐ দৃঢ়ভাবে একমত
- ☐ একমত
- ☐ নিরপেক্ষ
- ☐ অসম্মত
- ☐ দৃঢ়ভাবে অসম্মত

২৬. রোগীর চাহিদা-ই অ্যান্টিবায়োটিকের অতিরিক্ত ব্যবহারের জন্য দায়ী

- ☐ দৃঢ়ভাবে একমত
- ☐ একমত
- ☐ নিরপেক্ষ
- ☐ অসম্মত
- ☐ দৃঢ়ভাবে অসম্মত

২৭. কোন রোগীকে অ্যান্টিবায়োটিক দেবার সময় অ্যান্টিবায়োটিক প্রতিরোধ বিষয়টিও বিবেচনা করা দরকার।

- ☐ দৃঢ়ভাবে একমত

- ☐ একমত
- ☐ নিরপেক্ষ
- ☐ অসম্মত
- ☐ দৃঢ়ভাবে অসম্মত

২৮. আমি মনে করি যে,অপ্রয়োজনে রোগীদের অ্যান্টিবায়োটিক দেওয়া ক্ষতিকর।

- ☐ দৃঢ়ভাবে একমত
- ☐ একমত
- ☐ নিরপেক্ষ
- ☐ অসম্মত
- ☐ দৃঢ়ভাবে অসম্মত

২৯. আমি অ্যান্টিবায়োটিক প্রেসক্রিপশন সম্পর্কিত শিক্ষামূলক কর্মসূচী গ্রহণ করতে চাই।

- ☐ দৃঢ়ভাবে একমত
- ☐ একমত
- ☐ নিরপেক্ষ
- ☐ অসম্মত
- ☐ দৃঢ়ভাবে অসম্মত

৩০. অ্যান্টিবায়োটিক প্রতিরোধের বিরুদ্ধে লড়াই করার জন্য আমিও আমার কর্মক্ষেত্রে কিছু করতে পারি

- ☐ দৃঢ়ভাবে একমত
- ☐ একমত
- ☐ নিরপেক্ষ
- ☐ অসম্মত
- ☐ দৃঢ়ভাবে অসম্মত

৩১. অ্যান্টিবায়োটিক প্রতিরোধ বিশ্বব্যাপী একটি সমস্যা

- ☐ দৃঢ়ভাবে একমত
- ☐ একমত
- ☐ নিরপেক্ষ
- ☐ অসম্মত
- ☐ দৃঢ়ভাবে অসম্মত

৩২. অ্যান্টিবায়োটিক প্রতিরোধ আমার দৈনন্দিন কাজের ক্ষেত্রে একটি সমস্যা

- ☐ দৃঢ়ভাবে একমত
- ☐ একমত
- ☐ নিরপেক্ষ
- ☐ অসম্মত
- ☐ দৃঢ়ভাবে অসম্মত

৩৩. অ্যান্টিবায়োটিক দেবার সিদ্ধান্ত নেবার সময় কতবার আপনি আপনার সহকর্মীর সাথে আলোচনা করেন?

- ☐ কখনোই না
- ☐ কখনো কখনো
- ☐ বেশীরভাগ সময়
- ☐ সবসময়

৩৪. আপনি কতজনকে আপনার কর্মক্ষেত্রে অ্যান্টিবায়োটিক লিখে দেন?

- ☐ কাউকে না
- ☐ কয়েকজন রোগীকে
- ☐ বেশিরভাগ রোগীকে
- ☐ সকল রোগীকে

৩৫. সর্দি বা গলা ব্যথার জন্য কতজনকে অ্যান্টিবায়োটিক দেন?

- ☐ কাউকে না
- ☐ কয়েকজন রোগীকে
- ☐ বেশিরভাগ রোগীকে
- ☐ সকল রোগীকে

৩৬. একটি রোগীকে অ্যান্টিবায়োটিকের সম্পূর্ণ কোর্স দেওয়া হয়েছে। তিনি 3-4 টি নেওয়ার পর সুস্হবোধ করলে এটি নেওয়া বন্ধ করে দেন। আপনি কি এটা সঠিক মনে করেন?

- ☐ দৃঢ়ভাবে একমত
- ☐ একমত
- ☐ নিরপেক্ষ
- ☐ অসম্মত
- ☐ দৃঢ়ভাবে অসম্মত

৩৭. অ্যান্টিবায়োটিকের যথাযথ ব্যবহার সম্পর্কে আপনি রোগীদের কি কখনো বোঝান?

- ☐ কখনোই না
- ☐ কখনো কখনো
- ☐ বেশীরভাগ সময়
- ☐ সবসময়

৩৮. কতজন রোগী আপনার ক্লিনিক/ফার্মাসি-তে এসে অ্যান্টিবায়োটিক চান?

- ☐ কেউ না
- ☐ কয়েকজন রোগী
- ☐ বেশিরভাগ রোগী
- ☐ সকল রোগী
